# Care coordination of multimorbidity: a scoping study

**DOI:** 10.15256/joc.2015.5.39

**Published:** 2015-04-16

**Authors:** Anne Doessing, Viola Burau

**Affiliations:** ^1^VIA University College, School of Continuing Education, Centre for Leadership and Organisation Development (CLOU), Aarhus, Denmark; ^2^CFK – Public Health and Quality Improvement – Centre for Research and Development in Social and Health Services, Aarhus, Denmark; ^3^Department of Political Science and Government, University of Aarhus, Aarhus, Denmark

**Keywords:** Multimorbidity, care coordination, integrated care, chronic disease, disease management, complexity

## Abstract

**Background:**

A key challenge in healthcare systems worldwide is the large number of patients who suffer from multimorbidity; despite this, most systems are organized within a single-disease framework.

**Objective:**

The present study addresses two issues: the characteristics and preconditions of care coordination for patients with multimorbidity; and the factors that promote or inhibit care coordination at the levels of provider organizations and healthcare professionals.

**Design:**

The analysis is based on a scoping study, which combines a systematic literature search with a qualitative thematic analysis. The search was conducted in November 2013 and included the PubMed, CINAHL, and Web of Science databases, as well as the Cochrane Library, websites of relevant organizations and a hand-search of reference lists. The analysis included studies with a wide range of designs, from industrialized countries, in English, German and the Scandinavian languages, which focused on both multimorbidity/comorbidity and coordination of integrated care.

**Results:**

The analysis included 47 of the 226 identified studies. The central theme emerging was complexity. This related to both specific medical conditions of patients with multimorbidity (case complexity) and the organization of care delivery at the levels of provider organizations and healthcare professionals (care complexity).

**Conclusions:**

In terms of how to approach care coordination, one approach is to reduce complexity and the other is to embrace complexity. Either way, future research must take a more explicit stance on complexity and also gain a better understanding of the role of professionals as a prerequisite for the development of new care coordination interventions.

## Introduction

Despite the extent and impact of multimorbidity, most healthcare systems are organized within a single-disease framework, which does not reflect the problems and needs associated with multimorbidity [1–4]. The needs of patients with multimorbidity are not just the sum of the needs in relation to individual diseases [5], and, therefore, the single-disease organization has a negative effect on the continuity of care. It creates siloes across sectors where patients with multimorbidity are connected to several clinical pathways that are not coordinated with each other. As a consequence, patients may be confused about who is responsible for particular aspects of service delivery, and interrelated problems may not be dealt with quickly enough or may be duplicated by different providers.

Patients with multimorbidity are more vulnerable to organizational fragmentation [2], which arises when providers restrict their responsibility for care delivery to the patient when present, ignoring overall coordination across time and/or sectors. More specifically, fragmentation is described as the breakdown in communication and collaboration in providing services to an individual: this results in deficiencies in timeliness, quality, safety, efficiency and patient-centredness [6].

In many countries, attempts have been made to integrate healthcare services – for example, by implementing changes based on the Chronic Care Model, which (on an organizational level) typically involves the implementation of mono-diagnostic clinical pathways across sectors and the recruitment of chronic disease case managers, but the resulting structures rarely integrate the actual delivery of patient care [6]. Instead, as integration remains at the organizational level, providers often fail to fundamentally alter the manner in which healthcare professionals collaborate with each other, both within and across organizations, to coordinate their services [6]. At the same time, healthcare professionals in the secondary health sector have continuously moved towards specialization, which creates professional “siloes” [1]. This is problematic when a growing number of patients suffer from multimorbidity. Consequently, healthcare professionals themselves need to rethink and alter the way in which they work in order to reflect the needs and problems of patients with multimorbidity. In parallel, policy-makers must support and provide opportunities for such changes [1].

The definition of care coordination in this study follows the definition of McDonald et al. [7] based on a literature search and identification of key elements: *“Care coordination is the deliberate organization of patient care activities between two or more participants (including the patient) involved in a patient’s care to facilitate the appropriate delivery of health care services. Organizing care involves the marshalling of personnel and other resources needed to carry out all required patient care activities, and is often managed by the exchange of information among participants responsible for different aspects of care”* (McDonald et al., 2007. p. 3) [7].

The aim of care coordination is to facilitate the delivery of the appropriate healthcare services at the right time, in the right order, and in the right setting [7]. Care coordination is particularly critical when numerous healthcare professionals are involved in patient care. In such situations, there is a high level of interdependence among healthcare professionals who provide multiple services, making it necessary for them to have adequate knowledge about their respective roles to ensure exchange of information in a timely manner [7, 8].

In summary, it is important to know how care is effectively coordinated in relation to patients with multimorbidity and the implications for change that this will have for the provider organizations and the healthcare professionals who are responsible for care coordination.

We define multimorbidity as “multiple co-occurring chronic or long-term diseases or conditions, including both physical and mental diseases, and none considered as index disease”, as this is the most frequently used general definition [9].

The aim of this study was to determine the characteristics and preconditions of care coordination for patients with multimorbidity and the factors that promote or inhibit the development of care coordination among provider organizations and healthcare professionals. The study aimed to address these issues based on a literature review, which identified key themes to structure the summary of research findings.

## Methods

A scoping study was chosen for two reasons. First, the study of care coordination includes many different study designs and the strength of a scoping study lies precisely in its ability to account for variety and to produce results that are both broad and indepth [10, 11]. Second, and more generally, the scoping study is an approach to reviewing literature that aims to map the key concepts underpinning a research area and the main sources of evidence available [11]. This is particularly relevant as the existing research in this field is structured according to a range of overlapping terms and definitions. The scoping study combines a systematic literature search with a qualitative thematic analysis, similar to a narrative review. The specific methodology of this study followed the steps described by Arksey and O’Malley [11], with the exception of the optional element of consultation. This involved identifying relevant studies, study selection, charting the data and, finally, collation, summarizing and reporting the results.

### Identifying and selecting relevant studies

The search for relevant studies is described in Table 1. As neither multimorbidity nor care coordination is indexed in the selected databases, a free text search was used. The free text search was combined with an index search on the MeSH term “comorbidity/organization and administration”, and variations of care coordination were expanded by the term “integrated care”. The underlying rationale was to use neutral terms that are not linked to a linear notion of organizations; concepts such as shared care and case management were therefore not included. As multimorbidity, multidisease, multiple chronic diseases and comorbidity are sometimes used synonymously, the searches included all terms. The selection of search terms was based on an initial reading of relevant sources and an initial search of databases. Here, our concern was to find a balance between ensuring comprehensiveness of the search results and their manageability. From these initial readings/searches, we chose those terms that we assessed to give the most specific results.

The search strategy covered a 10-year period owing to the relatively recent recognition of multimorbidity as a concept in healthcare science. The search also only covered industrialized countries to ensure the comparability of the included studies. All searches in the databases were assisted by an experienced librarian.

### Data charting, collation and summarizing

*Charting* describes a technique for synthesizing and interpreting data by sifting and sorting material according to key themes and is akin to a narrative review [11]. The studies included were all read in full by the first author and then the first author recorded information from all included references in a data charting form due to standardized aspects, as illustrated in Table 2. Following this, a qualitative thematic analysis was conducted. Both authors carefully read through the extracted material with attention to central themes. After comparing the themes that were individually identified by both authors, we jointly selected the final themes of the study; this included three main themes with several sub-themes. Both authors then *collated* the extracted data into the final main and sub-themes, re-read the data and materials and *summarized* them.

## Results

**Figure 1:**
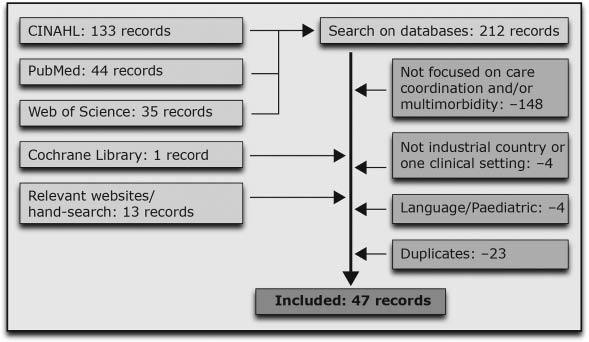
Flow diagram of study selection.

The searches in the databases generated 212 references, of which 179 were excluded because they were either duplicates or did not meet the selection criteria. Most were excluded because they did not include both care coordination and multimorbidity. A subsequent search on relevant websites, in the Cochrane Library, and in reference lists of relevant sources, generated an additional 14 references (Figure 1). The studies included were all read in full by the first author.

A total of 47 references were included in the scoping study (Table 3). Of these, there were three systematic reviews, 20 primary studies (ten quantitative, nine qualitative and one mixed method design), three research reports, 15 overview articles/literature reviews, five editorials/discussion papers and one position paper. The articles originated from the US, Canada, Australia, New Zealand and nine European countries. The perspectives of the studies covered the patient, the organization and the healthcare professional, and there was a tendency to focus on primary healthcare and older patients with multimorbidity.

The scoping study was based on two areas of interest – (i) the characteristics and preconditions of care coordination for patients with multimorbidity; and (ii) the factors that promote or inhibit care coordination at the levels of provider organizations and healthcare professionals.

In relation to both areas of interest, the overall theme that emerged from the scoping study was complexity: multimorbidity is complex medically, in terms of identifying/diagnosing and treating patients; and multimorbidity is complex organizationally, in terms of organizing the delivery of services at the levels of provider organizations and healthcare professionals. According to de Jonge et al. [20], the first dimension can be understood as case complexity and relates to the specific characteristics of patients with multimorbidity, whereas the second dimension concerns care complexity and is about healthcare service delivery. This distinction was chosen as a suitable, overall framework for the themes that we initially identified, although our study differs in one important respect. Whereas de Jonge and colleagues were specifically concerned with multimorbidity, we focused more specifically on multimorbidity and care coordination. This creates an interdependency among the dimensions that blurs the distinction between case and care complexity. In practical terms, we started by differentiating care complexity according to level and distinguished between three main types of complexity: case complexity, care complexity at the organizational level, and care complexity at the professional level. Subsequently, we integrated the initial themes that we had identified under each of the three types, as described in Table 4.

Case complexity is first and foremost about the *characteristics of patients with multimorbidity*; the combination of diseases makes patients a highly heterogeneous group, which is also difficult to categorize. We add an ‘insider view’ of case complexity by including *patient experiences with care coordination*; here, contacts with multiple healthcare professionals and the lack of care coordination are key.

Under care complexity at the organizational level, a first sub-theme is the relative *de facto* and desired *integration of individualized approaches* into a healthcare system based on standardized guidelines. Integration in itself is not sufficient, but care coordination also depends on *broader structures of healthcare*. Furthermore, as healthcare professionals are central for delivery, there is a *close connection between coordination at organizational and professional levels*.

Finally, care complexity at the professional level, in the first instance, includes the specific *roles in care coordination* that healthcare professionals can play. Building on this, *collaboration and communication* describes the specific forms of care coordination at this level.

## Case complexity

### Characteristics of patients with multimorbidity

Chronic diseases are progressive by nature, but there are huge variations in the way in which chronic diseases develop, how they are combined and how they impact the patient. In addition, the same combination of diseases may have different implications for different patients’ quality of life due to variations in psychosocial issues [4, 14, 16, 19, 23]. Factors such as gender and age also seem to have an influence on the implications of multimorbidity [18].

Furthermore, patients with multimorbidity are typically in contact with several healthcare professionals in both primary and secondary healthcare, which increases complexity [5, 12, 20, 23, 24, 47]. Even though many diseases usually do not require specialist treatment, the combination of diseases increases the burden of illness and leads to an extensive use of specialist services [55]. This is supported by McCormick et al. who state that, in hospice care, it is not one terminal condition that makes patients with multimorbidity ill, but rather the occurrence of multiple conditions at the same time [32].

Patients with multimorbidity are described as a heterogeneous group and this underlines both the need for and the difficulty of developing standardized methods to categorize different groups of patients. There are, however, examples of both category and categorization instruments.

One category of multimorbidity is described as “high impact multimorbidity”, which is defined as “*A debilitation combination of conditions that have a high impact* *on their own lives but also on their utilization of health services*” (Smith et al., 2012. p. 3) [48].

Another study used a simple morbidity score to categorize patients. The morbidity score was calculated from the number of chronic conditions and the self-reported healthcare status. Patients with high morbidity scores reported less favourable experiences with care coordination compared to those with a low morbidity score. In the same study, patients with multimorbidity were divided into groups with concordant and disconcordant conditions. This appears to be an important categorization as there was a statistically significant difference between the two groups because patients with concordant conditions had more favourable views about the coordination and quality of care than those with disconcordant combinations [18].

An example of a comprehensive categorization instrument is the INTERMED, which is an interview-based tool to identify complex patients who are in need of integrated care. It assesses case complexity by evaluating biopsychosocial health risks [29, 51].

### Patient experiences with care coordination

As patients with multimorbidity are typically in contact with multiple healthcare professionals, they have a higher risk of care coordination problems [5, 12, 30, 44]. Patients with three or more chronic conditions have roughly 25–40% greater odds of reporting care coordination problems than those with a single condition [30]. In addition, the likelihood of patients experiencing coordination problems increased sharply for patients seeing four or more physicians in all eight countries in the study by Schoen et al., whereas – across countries – the percentage of reported errors at least doubled among patients seeing four or more physicians compared to those seeing only one or two [44].

Patients with multimorbidity experience a number of specific challenges related to care coordination. These involve: standardized care plans that do not match their needs; different care plans that are in conflict with each other or too complex; inconsistent information; healthcare professionals who focus on their own clinical specialty rather than on the patient’s overall situation; and healthcare professionals who do not communicate with each other. Consequently, patients feel that they are sent from one specialist to another, consultations are overlapping and that they are forced to explain their symptoms over and over again [5, 13, 31, 34, 36]. According to Noël et al., most of the identified problems are not unique to patients with multimorbidity [36]. Multimorbidity, however, seems to magnify these problems or increase the probability that such problems will occur [17, 36]. The consequence of this is that “*Patients with multiple illnesses carry not only the burden of their illnesses, but also the burden of their multiple treatments”* (van der Vlegel-Brouwer, 2013. p. 2) [54].

Based on their experiences, patients with multimorbidity have a strong desire for care coordination across different healthcare and provider organizations. Patients’ perceptions of continuity of care are complex and typically associated with relational continuity, consistent information, connection between different initiatives and *ad hoc* access to relevant healthcare professionals [13, 31, 36].

Most of the patients with multimorbidity also expect to be involved in the coordination of their own care and they feel that they know their symptoms and needs well, although it is not always clear to them which symptoms relate to which diagnoses [26, 31]. However, not all patients wish to be involved in care coordination. This seems to apply particularly to patients with cognitive problems, low health literacy and those who are not familiar with the healthcare system or who are simply not able to advocate for themselves [17, 26, 34].

Several of the included studies support the need for an individual assessment and stress the patients’ need for a holistic focus in the coordination of their care. Ideally, this is based on an assessment that includes patients’ everyday life with multimorbidity, in addition to the clinical focus on managing or treating medical symptoms [5, 25, 40, 43, 45]. In general, patients’ subjective experience of care coordination can be an important input in the organization of healthcare [31]. The patient is often the only one to experience the entire pathway through the healthcare system, which gives them a unique position to evaluate care coordination. Patients can identify gaps or overlapping interventions or correct misinformation between healthcare providers. Patient evaluation of care coordination, can, therefore, guide the development of care coordination [31]. An important limitation in the use of patient evaluation, however, is that much care coordination is carried out behind the scenes without the patients’ knowledge. Therefore, it might be better to ask patients for problems rather than successes [30]. In fact, patients often expect care to be seamless, whereas flaws in care coordination are received as a bad surprise. Such problems can shake the confidence of patients in healthcare professionals and make them question the competence of healthcare professionals, leading to a potential non-adherence to treatment [26].

## Care complexity at the organizational level

### Relative integration of individualized approaches

The predominance of a linear and disease-specific organization of care delivery is embodied by the standardized approach of clinical guidelines [31, 46]. In clinical practice, healthcare professionals, to a large extent, use clinical guidelines to plan and document their services, thus increasing the standardization of care.

However, embracing case complexity at the level of organizations is important because the failure to do so may have negative consequences for the utilization of resources in the healthcare sector. In patients with multimorbidity, the use of guidelines developed for single diagnoses may lead to overtreatment and overly complex care regimes as the guidelines are considered to be proscriptive for best practice [40]. Similarly, Gilbert and colleagues show that quality indicators based on clinical guidelines can lead to unintended consequences when different diagnosis-related guidelines clash [24]. For the cited authors, this underlines the need to develop quality indicators based on patient preferences that are to be used in the evaluation of healthcare systems [24]. Erler et al. go even further and demand that the design of healthcare services should be based on patient needs: “*There is an urgent need to shift focus to the patient, whose need should form the starting point for designing care*” (Erler et al., 2011. p. 577) [22].

However, a study of the healthcare system in Quebec puts such claims into context. The provincial healthcare system has a long history of integrating healthcare services at the structural level, and a thorough assessment of the needs of patients is one of nine key conditions for integrating the delivery of healthcare services [53].

The literature widely acknowledges the need for a more holistic approach to care delivery for patients with multimorbidity [5, 55], whereas there are fewer suggestions as to what the specific implications are for the organization of care delivery. This is exemplified by a study by Bower et al. who found limited evidence that multimorbidity was actively considered in the organization of care, although general practitioners (GPs) and nurses were aware of the problems of patients with multimorbidity [17].

Concerning possible organizational change, one position is more minimalist and argues that acknowledging the need for individualized approaches to delivering healthcare is sufficient and that this is compatible with keeping diagnoses-related care programmes [5]. Another position is more maximalist and suggests that the care arrangements themselves need to be changed in order to be able to address the unique problems of patients and their shifting healthcare needs [13]. Decisions on care delivery must be multidimensional and, in terms of its specific organization, Bayliss et al. argue for introducing one single contact person together with a care concept that can respond to both continuing and changing needs [13].

Martin and Borst found that there generally is support for new approaches to healthcare delivery that build bridges between the day-to-day lives of patients on the one hand, and the healthcare system on the other [31]. Nevertheless, the order for change is demanding and such approaches face three more general organizational challenges: specialization, centralization and standardization [31]. Furthermore, responding to the specific suggestions of patients may be labour intensive. Instead, organizational interventions with a specific focus on a particular risk factor are likely to be more effective: “*Interventions to date have had mixed effects but have shown a tendency to improve prescribing and medication adherence, particularly if interventions can be targeted at risk factors of specific functional difficulties*” (Smith et al., 2012. p. 2) [48].

### The importance of broader structures of healthcare

Care coordination of patients with multimorbidity is also contingent on the broader structures of healthcare. Among these, the most important relates to the structures of healthcare delivery, both at the macro level of healthcare systems and at the meso level of healthcare providers [53]. In relation to the former, the relative structural differentiation of healthcare systems plays a significant role as they have become more complex with the emergence of new sub-disciplines and the availability of new treatments [5, 20, 50]. Organizing care delivery for patients with multimorbidity requires connections among different healthcare services [22, 38, 44, 45, 53]. This applies particularly to the coordination between the primary care sector and the secondary hospital sector [4]. A comparative study in eight countries found that “*Chronically ill patients in countries with strong primary care infrastructures tend to fare better. Yet deficits in transitional care when patients leave the hospital…exist in all countries*” (Schoen et al., 2009. p. 13) [44]. An underlying challenge is that the two sectors have very different approaches to care delivery: whereas in the secondary sector the diagnosis perspective is predominant, this is poorly compatible with the primary sector [41]. Therefore, any evaluation of new care coordination intervention must be designed to evaluate environmental influences [16, 25]. Smith et al. also recommend that multimorbidity interventions are integrated into existing healthcare systems for reasons of sustainability [48].

A related structural factor is financial incentives. The historical funding arrangements in many European countries pose significant barriers for organizing the delivery of healthcare services in an integrated and patient-centred way [4, 5, 56]. More specifically, the time needed for coordinating the delivery of healthcare services is typically not financially compensated.

Concerning the level of healthcare providers, several authors conclude that (overall) the substantial variations in the existing evidence regarding delivery system design, clinical settings, patient groups and quality measurement make it too early to draw firm conclusions regarding effectiveness of interventions [19, 21, 42, 48]. Nevertheless, studies identify leadership as an important factor: “*It is essential to have a leader – a ‘sense-maker in chief’ – who plays a critical role in shaping the direction of the current reform*” (Vedel et al., 2011. p. 7) [53]. This secures the long-term engagement in organizational change and supports the development of a common vision [25]. Leadership also includes network management [12]. The care delivery for patients with multimorbidity typically involves several treatment regimens that are located across different organizations. This has the character of a network, where individual organizations are often independent from each other, but where the relative success of care delivery depends on the collaboration with other organizations.

### Close connection between coordination at organizational and professional levels

As healthcare professionals are central for healthcare delivery, there is a close connection between coordination at organizational and professional levels. For example, among the factors that Bleijenberg and colleagues identify as promoting coordination among provider organizations, the majority relate to healthcare professionals [15]. These factors include training, involvement in developing organizational change, as well as good collaboration between doctors and nurses. Other studies found similar factors [28, 53]. Moreover, solid knowledge about other sectors among healthcare professionals, together with good communication, are important preconditions for strengthening the coordination between primary and secondary sectors [4]. Studies also highlight the importance of institutionalizing the coordination function – for example, in the form of multidisciplinary teams [14] or in case management [25, 43, 47]. However, Boult and colleagues caution that there are many conditions attached to the use of teams in terms of their structure, organization and governance, and teams may have greater effects in some patients than others [16]. Furthermore, models of integrated care delivery need to be supported by corresponding contractual arrangements, as well as by training [56]. With the proliferation of coordination institutions, there is also a need to harmonize the different coordinating functions – for example, by establishing a primary coordinator who acts as a ‘coordinator of coordinators’ [26, 38].

More generally, Implement found that the development of relations and trust across professionals working in different healthcare sectors was fundamental for securing the coordinated delivery of healthcare services for patients with multimorbidity [5]. Plochg and colleagues go even further and, in relation to the medical profession, claim that changes have to come from within the profession itself, although this can be supported by relevant policy pressure: “*Medical leaders, supported by health policy makers, can consciously activate the self-regulatory capacity of medical professionalism in order to transform the medical profession and the related professional processes of care so that it can adapt to the changing health needs*” (Plochg et al., 2009. p. 1) [39]. This is corroborated by another study, which found that nurses were quicker than doctors to adopt a collaborative team model, because the model better corresponded to the professional interests of nurses [52].

## Care complexity at the professional level

### Professional roles in care coordination

If healthcare professionals are to have an important role in the development of care coordination, as mentioned above, it is important to understand what role professionals play in care coordination. However, the analysis here suggests considerable uncertainty regarding the role of the various professions in care coordination. The same applies to the division of labor between specialists and generalists and between professionals in primary and secondary healthcare.

No one profession or sector is unanimously appointed as being primarily responsible for care coordination. However, a greater share of the sources focuses on primary healthcare [13, 15–17, 22, 27, 28, 34, 46, 48, 49, 52], which may indicate that this sector has had a special interest in care coordination.

In some studies, GPs are mentioned as having a key position in care coordination, although this is a new role involving more time spent on management [4, 15, 39]. At the same time, other studies question the engagement of primary care physicians in care coordination and suggest that other professionals – such as, for example, primary care nurses – should take over [4, 25, 33].

Likewise, some studies assign nurses a key role in care coordination [4, 5, 25, 37, 44]. For example, Boeckxstaens et al. argue that nurses in primary healthcare are ideally positioned to coordinate care delivery, as they spend more time with the patient and, thereby, have a better understanding of the patient’s expectations and needs [4]. Goodwin et al., however, question whether care coordination is best handled by nurses and, instead, introduce a continuum from non-clinical care coordinators to care coordination carried out by nurses involved in patient care [25]. A study by Schoen et al. found a significant variation across countries in the delivery of care coordination by nurses [44], and both GPs and patients criticized nurses for having insufficient clinical qualifications [15, 31, 41].

There is a need for a better understanding of the roles of generalists and specialists in managing patients with multimorbidity [48]. Generalists in primary healthcare meet patients in their own environment over a long period and have insights into patients’ medical and non-medical history, whereas specialists have a defined disease or organ focus and meet patients in short consultations [4]. Røsstad et al. found another difference – namely, that the focus in primary healthcare was on the patient’s functional abilities and social situation, whereas the focus in secondary healthcare was on evidence-based practice [41]. Not surprisingly, the professionals in the two sectors were struggling to understand each other and, for example, hospital nurses felt that primary care nurses were uninterested in the patient’s diagnosis and did not adhere to the specific guidelines for each disease. Conversely, primary care nurses found that specific guidelines did not match the complex needs of the patients, and often the primary care nurses did not receive sufficient information to carry out the necessary follow-up [41]. The challenge associated with sharing a common professional perspective is supported by Smith and Clarke [47] and Sinnott et al. [46] who describe how healthcare professionals such as GPs can be left with a sense of uncertainty regarding their role in their patients’ care: “*The involvement of multiple specialists and the emphasis on single disease care is antagonistic to the ‘holistic’ goals of GPs. This problem is compounded by poor co-ordination and communication within the health services, leaving GPs feeling excluded from their patients’ care and with a sense of uncertainty regarding their role*” (Sinnott et al., 2013. p. 8) [46].

Regardless of profession, the ultimate goal of the professionals is to improve the health of their patients through well-orchestrated, considerate and humane interventions [53]. Although many difficulties are logistical [46], the role of any care coordinator is far more complex than simply navigating people among care providers [25].

### The importance of collaboration and communication

Following on from the different roles that healthcare professionals can play in care coordination, collaboration and communication emerge as being key [4, 24–27, 35, 37, 38, 41, 47, 49, 53]. Poor collaboration is associated with substandard care as healthcare professionals need to have relevant information about the patient in order to coordinate actions with other providers in a complementary and timely manner [24, 26]: “*Connectedness matters for healthcare professionals because it translates into technical quality of care and patient safety”* (Haggerty, 2012. p. 1) [26].

In relation to multimorbidity, efficient collaboration is essential because no profession can manage such patients on their own. Face-to-face contact, a positive relationship and trust between professionals are important in order to achieve successful collaboration, including meaningful conversations about the complex needs of the patients [14, 24, 25, 41].

The current level of communication among healthcare professionals in different sectors seems to be insufficient, which is illustrated by Olsen et al. who found that nursing admission notes were present in only 1% of patient transfers from home care to the hospital, whereas 69% of patient discharges from the hospital to home care were accompanied by nursing discharge notes [37]. They found only one instance out of the 102 patients in their sample, in which a nursing transfer document was exchanged both at admission and at discharge [37]. In general, a lack of shared records and geographical distance are mentioned as barriers to communication [25, 38, 41].

A challenge to collaboration is that building up social networks among healthcare professionals in different organizations is perceived as a time-consuming activity that involves cultural and identity changes [12, 47, 53]. Also, the introduction of new staff – such as case managers – may disrupt existing communities of practice and can be perceived in a negative light in areas where good working relations among professions already exist [4]. Due to the many possible combinations of conditions in multimorbidity, the relevant professionals in the teams vary from patient to patient and the logistics of such *ad hoc* teams require a clear framework for collaboration and communication: “*Although the requirement to reflect on what sort of interprofessional team they are, and how they should operate is particularly great in such a case, the logistics are daunting and usually preclude it. …a ready framework on which to base their collaboration is required*” (Smith and Clarke, 2006. p. 538) [47].

## Discussion

### Strengths and weaknesses

The present scoping study contributes to the existing literature, with its combination of patient, healthcare professional and organizational perspectives. However, the results of our study have to be seen against the background of the more general strengths and limitations associated with conducting this type of literature review. A scoping study forces researchers to prioritize certain aspects of the literature, which requires reviewers to have high degrees of analytical skills [11]. The strength in this study is that both authors are experienced analysts and that they have different professional backgrounds. This helped to increase the validity of the analysis as identification of themes and summarizing were subject to mutual criticism. At the same time, close collaboration between the two authors allowed them to efficiently analyse data material on which they had different opinions. Another strength is that the included references comprise both quantitative and qualitative research, which is consistent with the specific characteristic of scoping studies.

One challenge with a scoping study such as the present study is that there is no appraisal of the quality of the included evidence [11]. This is significant because the identified themes are based on a variety of studies that may include more or less bias, but it is not possible to present a view regarding the ‘weight’ of evidence in relation to particular interventions. In the present analysis, the included studies showed considerable variation – for example, in relation to focus and country – and this gave a good opportunity for triangulation; namely, to contextualize individual studies and thereby to mitigate against possible bias. In general, the individual studies were of good quality and there was a high degree of consistency in the conclusions. Taken together, this leads us to consider the results of this study as robust.

Another methodological challenge is the unclear definition of multimorbidity and care coordination. This makes it difficult to clearly delineate the field of empirical studies and, by extension, to achieve data saturation. During the analysis, additional terms that overlap with care coordination were identified – for example, service organization and clinical management – and repeating the search with those terms might identify potentially relevant papers. Consequently, the results of this scoping study cannot be considered to be exhaustive.

### Care coordination in the context of complexity

Taking these limitations into account, where does this leave the care coordination for patients with multimorbidity? Or, in other words, how can we approach care coordination in the contexts of complexity? One approach is to reduce complexity. This is more or less implicit in discussions about the terminology used in relation to multimorbidity. Several studies [33, 48, 55] observe that there is considerable lack of clarity about the terms “multimorbidity” and “continuity of care”, reflecting the specific bodies of knowledge of different professions. Walker concluded that this makes it difficult to be certain about the prevalence of multimorbidity [55]. The underlying suggestion is that it is both possible and desirable to find a common definition. Another example is the discussion about the relationship between case and care complexity. The argument is that a precise assessment of the case complexity of patients with multimorbidity is required in order to match with an appropriate level of care complexity [20]. The relationship between the two is seen as relatively linear and, indeed, Smith and Clarke [47] offer definitions of the two types of complexity, which are measurable in quantitative terms.

Another approach is to embrace complexity. For example, van der Vlegel-Brouwer and Soubhi et al. view care coordination for patients with multimorbidity as a complex dynamic system [49, 54]. Complex adaptive systems are based on two assumptions: first, that the skills for care coordination do not rest with one party, but are the result of cooperation involving multiple parties; and second, that change towards greater care coordination occurs incrementally in successive adaptations. Multimorbidity, in particular, strongly illustrates the complexity of care coordination, but this issue is also relevant for other areas of care coordination, as recent contributions to the literature illustrate. Based on a recent study of public health partnerships in England, Hunter and colleagues call for less formalized and strategic approaches to coordination [57]. Similarly, Tsasis and colleagues conclude that one possible explanation for the lack of organizational change towards integration of local healthcare networks in Ontario is that the healthcare system continues to be treated in a linear fashion [58]. Finally, in a more conceptual paper, Edgren argues that, although the machine metaphor has long shaped the view of an effective organization in healthcare services, it is inappropriate considering the changing needs and preferences of patients [59].

### Implications for future research

Considering its centrality for multimorbidity, future research on care coordination for patients with multimorbidity needs to take a more explicit stance on the issue of complexity in connection to the design, as well as the evaluation of interventions, to improve outcomes for patients with multimorbidity.

Based on the identified studies (Table 3), currently it seems inexpedient to conduct a full systematic review as there is insufficient evidence to assess the effectiveness of specific care coordination interventions in multimorbidity among provider organizations and healthcare professionals. The most surprising gap in the identified literature is the lack of analyses of the role of healthcare professionals in care coordination. It is not clear what specific role different healthcare professionals have in care coordination, how they understand their own role, and what they do when they are coordinating care in multimorbidity. However, this seems to be a prerequisite for the development of care coordination interventions, especially if healthcare professionals are to play an active role in these interventions, and change has to come from within the professions, as suggested by Plochg and colleagues [39]. Consequently, future research on the roles of the professionals involved in care coordination is best made prior to the development and empirical tests of new care coordination interventions in multimorbidity.

## Conclusion

Multimorbidity is a key challenge for healthcare systems worldwide, and there is a large number of patients who suffer from multimorbidity. As this scoping study demonstrates, this challenge revolves around complexity and the following three themes.

First, case complexity, which encompasses the characteristics of patients with multimorbidity and patient experiences with care coordination. Medically, patients with multimorbidity have many diseases, which can occur in a limitless number of combinations. This not only increases the number of providers involved and the risk of care coordination problems but also makes it more difficult to develop standardized methods to categorize different groups of patients. Patients’ experiences of care coordination come with their own sets of complexities, not least because most patients expect to be involved in care coordination.

Second, care complexity at the organizational level, which includes several sub-themes. The relative integration of individualized approaches highlights the fact that the linear and disease-specific organization of care delivery based on guidelines potentially limits the possibilities for tailoring care delivery to individual patients. However, there are different views about how strong the trade-off is. Another sub-theme concerns the importance of broader structures of healthcare – the organization of care delivery for patients with multimorbidity is contingent on a complex set of structural factors. These are located at the macro level of healthcare systems, as well as at the meso level of healthcare providers.

The close connection between coordination at organizational and professional levels further complicates organizing the delivery of care. Ideally, coordination requires ownership by the healthcare professionals involved and cannot necessarily be imposed on them. And, third, this is exacerbated by care complexity at the professional level. This is because there is uncertainty about professional roles in care coordination. Furthermore, as no profession can manage patients with multimorbidity on their own, the importance of collaboration and communication among healthcare professionals is high. Currently, communication across sectors is insufficient and it is a challenge to build up the necessary social networks among healthcare professionals in different organizations.

Interventions directed at patients with multimorbidity can either reduce or embrace complexity. Either way, future research must take a more explicit stance on complexity and also gain a better understanding of the roles of professionals as a prerequisite for the development of new care coordination interventions.

## Figures and Tables

**Table 1:** Search strategy.

Search terms	Multimorbid* OR multi-morbid* OR multidisease OR multi-disease OR “multiple chronic diseases” OR comorbidity (CINAHL headings), comorbidity/organization and administration (MeSH) AND coordination OR co-ordination OR “coordination of care” OR “integrated care”
Timespan	2003–2013
Time of search	November 2013
Sources	Databases: • PubMed, CINAHL, Web of Science The Cochrane Library Websites of relevant networks and organizations: • Danish health authorities, regional and municipal institutions in Denmark, The Kings Fund, National Institutes of Health, International Research Community on MultimorbidityHand-search of reference lists
Inclusion criteria	Languages: • Danish, English, German, Swedish, Norwegian Sources from Denmark and other industrial countries
Exclusion criteria	Sources that only focus on coordination within one clinical setting

**Table 2:** Data charting form.

Data were recorded based on the following aspects:	
•	Authors, year of publication, type of publication
•	Study location, study population
•	Aims
•	Characteristics and preconditions of care coordination in patients with multimorbidity
•	Factors promoting or inhibiting progress of care coordination among healthcare professionals
•	Factors promoting or inhibiting progress of care coordination among provider organizations
•	Other important results

**Table 3:** List of included records.

Study/Reference	Country	Population/Theme	Type of publication/design	Purpose
Amelung and Wolf, 2011 [12]	Germany	Patients with multiple chronic diseases	Overview article	Not described explicitly
Bayliss et al., 2008 [13]	USA	Elderly patients with multimorbidity in community housing	Primary study/qualitative interviews	Explore processes of care desired by elderly patients
Berry et al., 2013 [14]	USA	Patients with complex health profiles/integrated care coordination program	Primary study/institutional case study	Not described explicitly
Bleijenberg et al., 2013 [15]	Netherlands	GPs and nurses	Primary study/survey	Report expectations and experiences of U-CARE programme
Boeckxstaens et al., 2011 [4]	Europe	Primary care	Position paper	Address the needs of older patients in primary care practice
Boult et al., 2011 [16]	USA	Older patients with multimorbidity	Primary study/cluster-RCT	Measure effect of guide care teams on use of healthcare services
Bower et al., 2011 [17]	UK	Patients with multiple long-term health conditions/primary care	Primary study/qualitative interviews	Explore GP and nurse perceptions of multimorbidity and influence on service organization and decision-making
Burgers et al., 2010 [18]	Australia, Canada, France, Germany, Netherlands, New Zealand, UK, USA	Chronically ill adults from eight countries with and without multimorbidity	Primary study/telephone survey	Examine whether experience of patients varies in terms of coordination of care and overall quality
De Bruin et al., 2012 [19]	Netherlands	Patients with multiple chronic conditions/comprehensive care programmes	Systematic literature review	Provide insights into characteristics of comprehensive care programmes and their impact
De Jonge et al., 2006 [20]	Netherlands	Complex patients who have comorbidities	Overview article	Evaluate the potential use of concept of complexity
DuGoff et al., 2013 [21]	USA	Multimorbidity/care coordination quality measures	Primary study/systematic search and assessment	Identify what care coordination processes are being measured and assess alignment
Erler et al., 2011 [22]	Netherlands, USA, UK	Primary care systems	Primary study/analytical comparative study	Analyse main problems for primary care and review strategies and practice models
Füsgen, 2011 [23]	Germany	Geriatric patients including patients with multimorbidity	Overview article/discussion paper	Discussion of geriatric efforts, including structural changes
Gilbert et al., 2011 [24]	Australia	Older patients with multiple health problems	Primary study/database survey and interviews	Identify and evaluate management and care of multiple chronic health problems
Goodwin et al., 2013 [25]	UK	Care coordination programmess for people with long-term and complex chronic conditions	Report/comparative analysis	Examine key lessons and markers for success in the “how” of care coordination
Haggerty, 2012 [26]	Canada	Patients with multimorbidity and continuity of care	Editorial	Not described explicitly
Implement, 2013 [5]	Denmark	Patients with chronic conditions/primary and secondary healthcare	Report	Thematic description of the results and experiences from 234 government-funded projects
Jones and Rosenberg, 2011 [27]	USA	Integration and coordination across behavioural health and primary care	Primary study/survey	Identify how members gauged importance of integration
Kathol et al., 2010 [28]	USA	Integration of mental health intervention in primary care settings	Primary study/interviews	Assess pragmatic challenges of implementing, delivering and sustaining models of integrated care
Latour et al., 2007 [29]	Netherlands	Complex medically ill patients/the INTERMED	Overview article	Describe the INTERMED method and its applicability to nursing process
Maeng et al., 2012 [30]	USA	Adults with chronic conditions	Primary study/telephone survey	Identify factors associated with perception of care coordination problems
Martin and Borst, 2013 [31]	Denmark	Patients with COPD and multimorbidity and their caregivers	Primary study/interviews	Describe experiences of patient and caregivers in relation to continuity of care
McCormick and Boling, 2005 [32]	USA	Older patients with multimorbidity	Editorial	Not described explicitly
Mollica and Gillespie, 2003 [33]	USA	People with (multiple) chronic conditions	Report	Explore the components of care coordination and a sample of state initiatives
Newbould et al., 2012 [34]	UK	Elderly patients with long-term conditions	Primary study/interviews	Explore variations and emergent experience of care planning
Noël et al., 2007 [35]	USA	Patients with multimorbidity compared to patients with single chronic illnesses	Primary study/cross sectional survey	Examine self-management learning needs and willingness to see non-physician providers
Noël et al., 2005 [36]	USA	Primary care patients with multimorbidity	Primary study/interviews	Explore collaborative care needs and preferences
Olsen et al., 2013 [37]	Norway	Older patients who are transferred between health care organizations	Primary study/database survey	Evaluate prevalence of nursing transfer documents and identify patient and transfer characteristics
Paulus et al., 2013 [38]	Belgium	Management of chronic diseases/patients with multimorbidity	Overview article	Describe development and main stances for proposed reforms
Plochg et al., 2009 [39]	Netherlands	Professional organizations/medical professionals	Discussion paper	Changing medical professionalism to fit changing health needs of complex and chronically ill patients
Roland and Paddison, 2013 [40]	UK	Patients with multimorbidity/management	Overview article/analysis	Discuss current problems and suggest steps for improvement
Røsstad et al., 2013 [41]	Norway	Development of patient-centred care pathway across healthcare providers	Primary study/interviews and observation	Investigate process and experience of participants
Salisbury, 2013 [42]	UK	Multimorbidity	Editorial	Not described explicitly
Sampalli et al., 2012 [43]	Canada	Individuals with multimorbidity	Overview article and pilot results	Describe integrated model of care and results of pilot evaluation
Schoen et al., 2009 [44]	International	Adults with chronic conditions who had recent healthcare experience	Primary study/survey	Patient experience of access, coordination, safety and care management
Singer et al., 2011 [45]	USA	Object of care integration and essential components	Overview article	Propose definition and measures based on the definition
Sinnott et al., 2013 [46]	Ireland	Patients with multimorbidity	Systematic review and meta ethno-graphic synthesis	Synthesize the literature on GPs’ experiences of clinical management of multimorbidity
Smith and Clarke, 2006 [47]	Australia	Integrated interventions and chronic illness	Overview article	Review what is learned about integrated care and discuss conceptual and methodological difficulties
Smith et al., 2012 [48]	Ireland	Patients with multimorbidity in primary care and community settings	Cochrane Review	Determine the effectiveness of interventions designed to improve outcomes
Soubhi et al., 2010 [49]	Canada	Patients with multimorbidity	Overview article	Introduce primary care practice model
Stiefel and Huyse, 2006 [50]	Switzerland	Complex patients with biopsychosocial comorbidities/integrated care	Discussion paper	Not described explicitly
Stiefel et al., 2006 [51]	Switzerland	Operationalizing Integrated Care – The INTERMED Project	Overview article	Describe the INTERMED (interview-based instrument to assess case complexity)
Vedel et al., 2013 [52]	France	Collaborative care model in primary care	Primary study/longitudinal case study	Analyse the PCPs’ and nurses’ decision to adopt or not and to determine model’s diffusion process
Vedel et al., 2011 [53]	Canada	People with multiple chronic conditions in Quebec	Wide-ranging literature review	Describe the transformation underway and results of recent initiatives in integrated health and social care
van der Vlegel-Brouwer, 2013 [54]	Netherlands	Chronically ill/integrated care	Overview article	Not described explicitly
Walker, 2012 [55]	Australia	Multiple conditions	Qualitative literature review	Explore current literature
Wulsin et al., 2006 [56]	USA	Models of integrated care	Overview article	Describe models

COPD, chronic obstructive pulmonary disease; GP, general practitioner; PCP, primary care practitioner; RCT, randomized controlled trial.

**Table 4:** Main and sub-themes.

Main themes	Sub-themes
Case complexity	Characteristics of patients with multimorbidity
	Patient experiences with care coordination
Care complexity at the organizational level	Relative integration of individualized approaches
	Importance of broader structures of healthcare
	Close connection between coordination at organizational and professional levels
Care complexity at the professional level	Professional roles in care coordinationThe importance of collaboration and communication
